# Unraveling the effects of serial intravitreal Aflibercept injections on the ocular surface of patients with glaucoma and retinal comorbidity

**DOI:** 10.1038/s41598-025-98436-8

**Published:** 2025-04-15

**Authors:** Luca Agnifili, Maria Ludovica Ruggeri, Michele Figus, Luca V. Corboli, Matteo Fornaro, Giuseppe Covello, Rodolfo Mastropasqua, Marta Di Nicola, Annalisa Marotta, Leonardo Mastropasqua

**Affiliations:** 1 Ophthalmology Clinic, Department of Medicine and Science of Ageing, “G. d’Annunzio” University Chieti-Pescara, Chieti, 66100 Italy; 2 Department of Surgical, Medical, Molecular Pathology and Critical Care Medicine, University of Pisa, Pisa, 56126 Italy; 3 Ophthalmology, Department of Medical and Surgical Specialties, Azienda Ospedaliero Universitaria Pisana, Pisa, 56124 Italy; 4 Department of Neuroscience, Imaging and Clinical Science, “G. d’Annunzio” University Chieti- Pescara, Chieti, 66100 Italy; 5 Laboratory of Biostatistics, Department of Medical, Oral and Biotechnological Sciences, “G. d’Annunzio” University Chieti-Pescara, Chieti, 66100 Italy

**Keywords:** Glaucoma, Glaucoma therapy-related ocular surface disease, Retinal diseases, Anti-VEGF, Aflibercept, Intravitreal injections, Povidone-iodine, Prognostic markers, Retina

## Abstract

To evaluate ocular surface and eyelid modifications occurring in glaucomatous patients diagnosed with glaucoma therapy-related ocular surface disease (GT-OSD) and retinal comorbidities who previously underwent serial Intravitreal injections (IVIs) of aflibercept. Thirty-two eyes of 32 patients with a diagnosis of GT-OSD and concomitant retinal diseases were enrolled in a two-center retrospective observational study. The main outcome measures were: Noninvasive tear film break-up time (NIBUT), Tear meniscus height (TMH), Bulbar redness score (BRS), fluorescein Tear film Break Up Time (TBUT), Corneal Fluorescence Staining (CFS), Schirmer test I (ST), and inferior eyelid Meibomian Glands (MGs) dropout. Differences between treated and fellow eye (TE, FE), were considered. The median number of IVIs (aflibercept) in TE was 4 (interquartile range (IQR) 3-6.50). Mean BRS was significantly lower (*p* = 0.011) and median TBUT higher (*p* = 0.037) in TE compared to FE. Despite CFS and NIBUT did not significantly differ between eyes, their median values showed a marginal tendency for better results in TE compared to FE. Serial IVIs of aflibercept positively affected some features of the GT-OSD, reducing conjunctival hyperemia and improving the tear film stability. These preliminary results could open to new strategies for ocular surface management in glaucoma, whether confirmed in larger prospective studies.

## Introduction

Glaucoma therapy-related ocular surface disease (GT-OSD) is an imbalance of the ocular surface homeostasis induced by the chronic use of topical intraocular pressure (IOP) lowering medications, characterized by tear film instability, epithelial damage, and inflammation^1^.

Histopathologically, GT-OSD resembles a particular form of dry eye, affecting up to 59% of patients^1–7^. It negatively impacts different aspects of disease management since produces boring symptoms, reduces the patient’s quality of life (QoL) and adherence to therapy, worsens the control of the disease, and increases the risk of surgical failure^8,9^.

To date, there are no standardized approaches to contain the GT-OSD, although is recommended the use of preservative-free IOP lowering formulations, the therapy regimen simplification, the mitigation of dry eye and inflammation, and eyelid improvement^10,11^.

Griffith & Goldberg reported a higher prevalence of retinal diseases in patients with glaucoma, being the two conditions co-diagnosed in up to 14.8% of cases^12^. Moreover, a recent study that investigated the inflammatory features characterizing the ocular surface in patients with GT-OSD, reported a higher VEGF concentration in tears^13^.

In a recent study, Malmin et al. observed unexpected favorable effects on the ocular surface of patients with neovascular AMD (n-AMD) and dry eye, induced by the serial administration of intravitreal injections (IVIs) of anti-VEGFs^14^.

To date, no previous studies investigated whether serial IVIs of anti-VEGFs may induce similar changes on the ocular surface of patients with glaucoma and comorbid retinal diseases. In the present study, we retrospectively evaluated whether the administration of serial IVIs of anti-VEGFs in glaucomatous patients with GT-OSD and retinal comorbidities produced some favorable modifications of the ocular surface and eyelids.

## Materials and methods

A total of thirty-two eyes of 32 patients were enrolled in this two Italian Centers retrospective observational study. Patients receiving unilateral IVIs were enrolled at the Glaucoma and Retina services of the Ophthalmology Clinics of the University of Chieti-Pescara and Pisa, between January 2023 and May 2024.

This study was approved by the institutional review board of the Department of Medicine and Ageing Science of the University ‘G. d’Annunzio’ of Chieti-Pescara (Chieti, Italy) (IRB approval no. 2023.0083), and it complied with the principles of the Declaration of Helsinki. Before enrollment, patients were asked to sign an informed consent form that reported the nature and possible consequences of this research.

### Inclusion criteria

Patients aged 18 years or more, with a diagnosis of open-angle glaucoma, GT-OSD, and a retinal comorbidity were considered eligible for this study. In detail, an IOP < 18 mmHg (mean of three measurements taken at 9 AM, 12 noon and 4 PM) controlled with topical medications, a visual field test (Humphrey field analyzer II 750 (Carl Zeiss Meditec Inc., Dublin, CA) (24 − 2 test, full-threshold)) showing at least three contiguous points on the total deviation probability plot at the less than 2% level, Glaucoma Hemifield Test *outside normal limits* and ophthalmoscopic signs of glaucomatous optic disc consistent with the VF alterations, were required. The last visual field had to be performed at least four months before enrollment, otherwise, the examination was repeated on the day of enrollment.

Any topical IOP lowering therapy schedule was permitted (from one to four active compounds per day), but the therapy regimen had to be unmodified in the last four months before enrollment.

A concomitant diagnosis of GT-OSD was required. Given the similarity between GT-OSD and dry eye and the absence of standardized diagnostic criteria, the presence of GT-OSD was based on the TFOS DEWS II Diagnostic Methodology Report: ocular surface disease index (OSDI) score > 12, break-up time (BUT) < 10 s or corneal fluorescein staining (CFS) > 2, according to Oxford grading scale^1,9,15^. Finally, all glaucomatous patients had to be affected with retinal comorbidities, such as n-AMD, diabetic macular edema (DME), or retinal vein occlusion (RVO), with a history of at least 2 previous IVI in the study eye, and at least 4-weeks interval between the last IVI and the execution of the ocular surface tests.

### Exclusion criteria

patients with previous ocular surgeries (excluding cataract surgery performed more than 12 months before enrollment), concomitant ocular or systemic autoimmune disorders, history of dry eye or MGs dysfunction before the diagnosis of glaucoma, any additional topical medications excluding lubricants, history of hypersensitivity to intravitreal anti-VEGFs or their excipients, hypersensitivity to anti-septic eyedrops, previous IVI-related complications or administration of intravitreal steroids, concomitant eyelid disorders, facial nerve palsy, previous ocular trauma, contact lens wearing, end-stage glaucoma, were not considered eligible.

The number of previous IVIs received by patients was obtained from the patient electronic records. The fellow eye, affected with glaucoma and controlled with the same regimen of IOP lowering topical medications of the study eye, but without concomitant retinal disease, served as control.

### IVI procedure

All IVIs were performed in the operating room by two experienced surgeons (RM and MF). Before accessing the operating theatre, mydriasis of the study eye was obtained with the administration of two consecutive drops (10’ intervals) of unpreserved tropicamide 1% (Visumidriatic 1%, Visufarma S.p.A., Rome, Italy). Once in the operating theatre, all patients received one drop of topical unpreserved lidocaine hydrochloride 1% ophthalmic solution (Lidocaina Cloridrato Intes, Alfa Intes, Napoli, Italia). Afterward, povidone-iodine (PVP-I) 5% (Betadine, Alcon) was used to treat the ocular surface (one drop), and to obtain antisepsis of the peri-ocular skin, eyelid margin, and eyelashes. After the application of the eye speculum, and immediately before the IVI (0,05 ml of drug via pars plana using a 30G needle), one drop of PVP-I 5% was again administered on the ocular surface. Antiseptic washout with saline irrigation was not performed at the end of the procedure. Topical IOP lowering therapy for glaucoma continued unmodified either the same day of the IVI procedure or the following days.

## Clinical assessment and ocular surface tests

Immediately after enrollment, before initiating the clinical evaluation and the ocular surface assessment, the OSDI questionnaire was administered to evaluate the GT-OSD. Then (between 9 and 11 o’clock) patients underwent a complete ophthalmological examination of the anterior and posterior segments, including Goldmann applanation tonometry and undilated fundoscopy. To avoid any potential bias induced during the initial enrollment phase, the ocular surface assessment was performed in the early afternoon before the instillation of any other eyedrop.

**Fig. 1:**
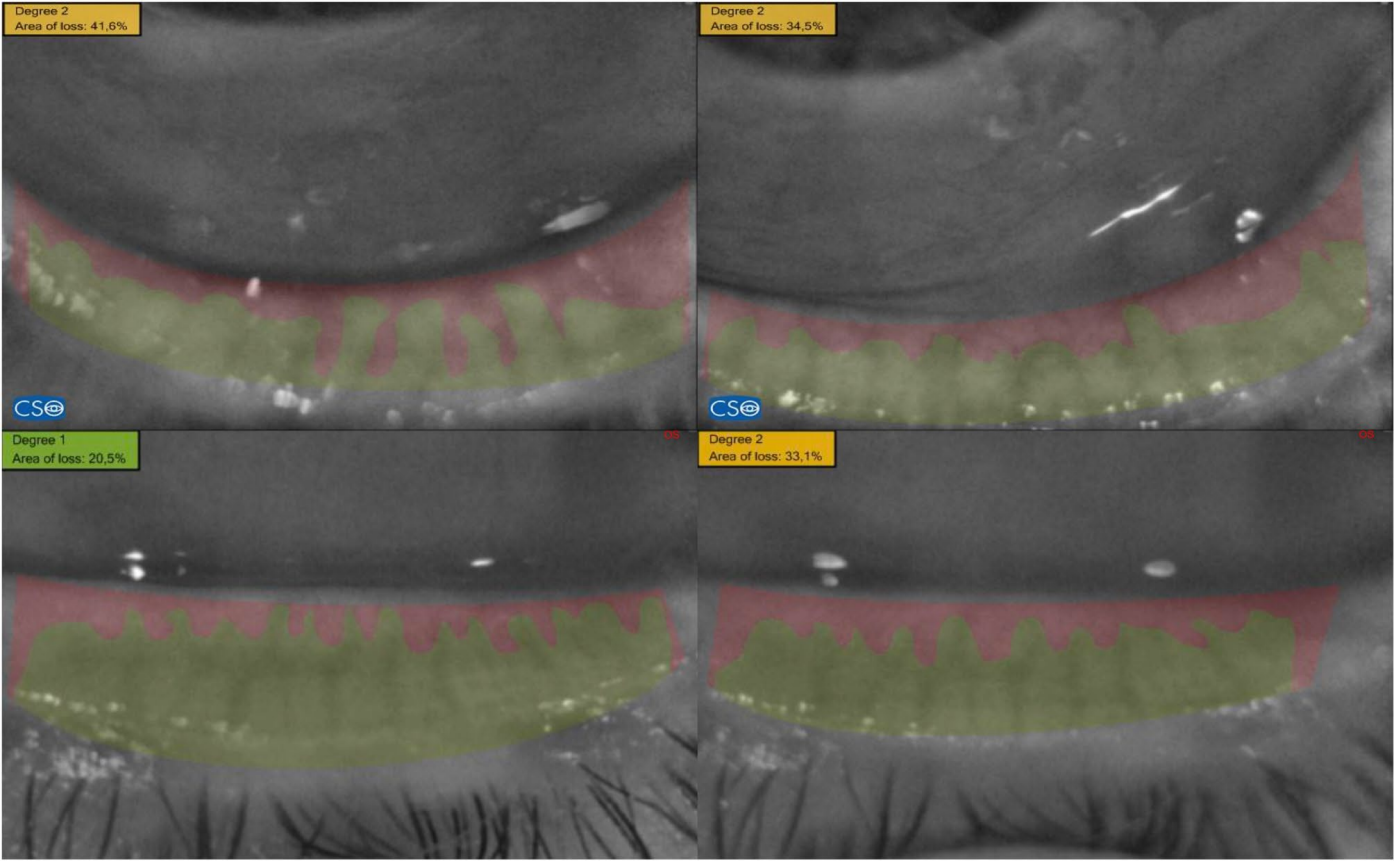
ImageJ-assisted meibomian gland dropout analysis. Mosaic of four meibographies in which the delimited green regions, manually marked, show the area covered by MGs. The dropout area is calculated as the difference between the unmarked and marked area of the tarsum.

Ocular surface tests were consequentially performed, from the least to most invasive test: (i) *noninvasive tear film break-up time (NIBUT)*, evaluated with the Sirius corneal topographer/tomographer system (Costruzione Strumenti Oftalmici (CSO), Florence, Italy). (ii) *Tear meniscus height (TMH)* was analyzed at the central portion of the inferior eyelid margin, by using the MS-39, a combined optical coherence tomography, and Placido Disk Topography device (CSO, Florence, Italy). *(iii) Bulbar redness score (BRS)*, a measure of the bulbar conjunctival hyperemia was graded using the EFRON score (0 as minimum score and 4 as severe, maximum score). (iv) *Tear film BUT (TBUT)* with fluoresceine was determined as the time interval between a blink and the first emergence of a dry spot on pre-corneal tear film (a value equal to or superior to 10 s was considered normal). (v) *CFS*was analyzed by using a biomicroscope with cobalt-filtered light. A small drop of fluorescein sodium 2% solution was applied to the inferior tarsus, and fluorescein staining of the cornea was evaluated according to the Oxford grading scheme ranging from 0 to 5^16^. *Schirmer test I (ST)*was performed without anesthesia using sterile strips, with normal range being considered values equal or superior to 15 mm. BUT, CFS, and (30 min after BUT and CFS measurements) Schirmer test I (STI) without topical anesthesia, were executed in the order suggested by the DEWS guidelines^15^. vii) *MGs dropout*: MGs were evaluated with Meibography by using the Sirius corneal topographer/tomographer system, to quantify the MG loss at the inferior eyelid. MG loss was expressed as *Area Dropout/Area Tarsal plate x100%*, and was determined by using the ImageJ software (Fig. 1). The operators (LC and MF) were masked with respect to the patient’s history and for the eye receiving treatment.

### Statistical analysis

Statistical analyses were performed using R software environment for statistical computing and graphics (version 4.2; http://www.r-project.org/). For quantitative variables, the median and interquartile range (IQR) are presented. Qualitative variables were described using the absolute frequency (n) and column percentage (%). Differences were tested for normality distribution using the Shapiro-Wilk test. Wilcoxon signed- rank test was used to detect if there were statistically significant differences between treated and fellow eye (TE, FE) for the studied variables. Boxplots have been used to graphically report statistically significant results. Spearman rank correlation coefficient has been used to analyze correlations between differences in ocular surface parameters, and different variables including age, number of intravitreal injections, duration of hypotonic treatment, and number of eyedrops. All statistical tests were two-sided, with a significance level set at *p* ≤ 0.05.

A post-hoc power analysis with respect to TMH (mm) as the primary outcome was conducted. Considering for a paired test an effect size of Cohen (d) of 0.60 our sample of 32 eyes would achieve 90% of power. We have set a significant level (α) at 0.05, correlation among the repeated measures (TE and FE) at 0.5 and non-sphericity correction εat 1.

## Results

The demographic and clinical characteristics of patients are summarized in Table 1. The median number of IVIs in treated eyes was 4 (IQR 3.20–6.60), whereas the median time interval between the most recent IVI and the ocular surface assessment was 4 weeks (IQR 4–8). All patients in the present sample received aflibercept 2 mg and followed a treat-and-extend protocol, entailing.

**Table 1:** Demographic, clinical characteristics, and IVI-related features of the enrolled patients.

Patients		
Total *(n*,* %)*	32	100
Male *(n*,* %)*	22	68.8
Age *(years)*,* median (IQR)*	76	(IQR, 59–78)
Systemic BBs/ARBs/CBBs/diuretics *(n*,* %)*	8/7/7/2	33, 29, 29, 9
Clinical features		
IOP *(mmHg)*,* median (IQR)*	14	(IQR, 13–16)
MD *(dB)*,* median (IQR)*	−5.87	(IQR, −3,91 - −7,83)
IOP lowering medications *(N)*,* median (IQR)*	2	(IQR, 1–2)
Topical PGAs/BBs/CAIs-BAK *(n*,* %)*	20/22/12–22	68/53/53–68
Duration of IOP lowering medical therapy *(years)*,* median (IQR)*	5	(IQR, 2.75–8)
OSDI score	36	(IQR, 22–51)
Patients using lubricants (sodium hyaluronate) *(n*,* %)*	32	100
Retinal comorbidity		
n-AMD *(n*,* %)*	18	56.2
DME *(n*,* %)*	7	21.9
RVO *(n*,* %)*	7	21.9
IVIs-related feature		
Patients receiving antiseptic drops* *(n*,* %)*	30	93.8
Number of antiseptic eyedrops* *(n*,* median IQR)*	1.5	(IQR, 1–2)
Number of IVIs *(n)*,* median (IQR)*	4	(IQR, 3.20–6.60)
Time interval most recent IVI *(weeks)*	4	(IQR, 4–8)
Cataract Surgery		
Yes *(n*,* %)*	22	68.8
No *(n*,* %)*	10	38.2

N: number; n-AMD: neovascular-age related macular degeneration; DME: Diabetic Macular Edema; RVO: Retinal Vein Occlusion; IOP: intraocular pressure; IVIs: intravitreal injections; MD: mean deviation; OSDI: ocular surface disease index; IQR: interquartile range expressed as first and third quartile; dB: decibel.

PGAs: prostaglandin analogs; BBs: beta-blockers; CAIs: carbonic anhydrase inhibitors; ARBs: angiotensin II receptor blockers; CCBs: calcium channel blockers.

* povidone-iodine 5%.

3 consecutive monthly injections until disease inactivity is recognized, followed by progressive extension of the treatment interval in increments of 2 to 4 weeks, up to a maximum interval of 12 to 16 weeks. Treatment intervals are then shortened when disease activity recurs. Significant IVI-related complications were not reported in any of the cases. All patients had controlled IOP, an early-to-moderate glaucoma (Hodapp-Parrish-Anderson criteria), and a moderate to severe form of GT-OSD at enrollment^17^.

**Fig. 2:**
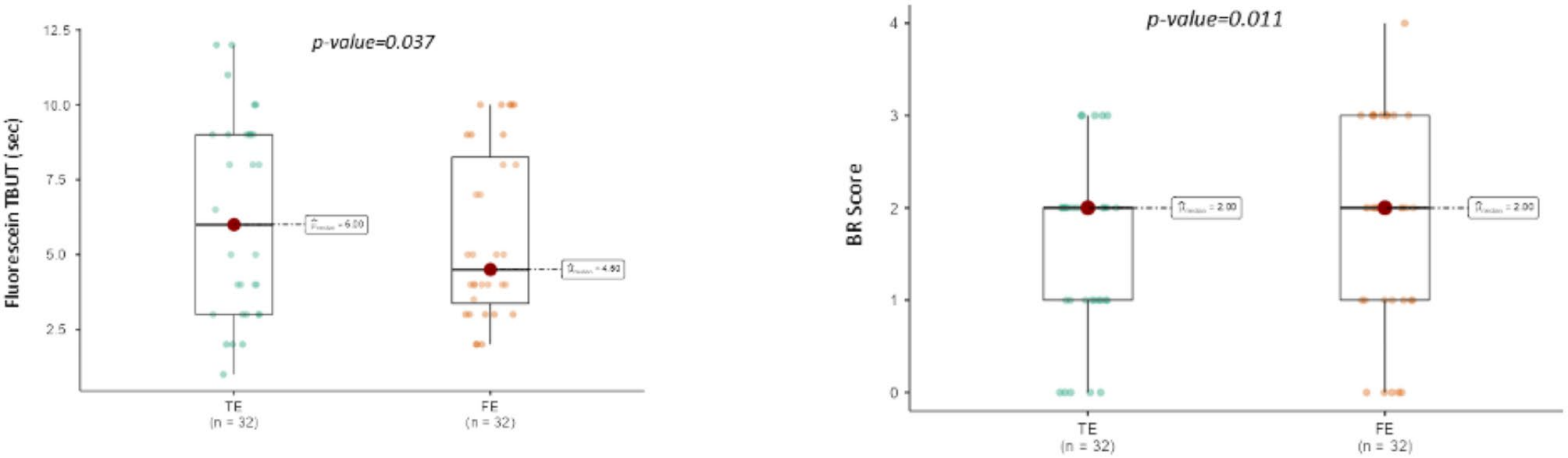
Differences between treated and fellow eyes regarding bulbar redness score (BRS, left), tear film break-up-time (TBUT, right). Boxes represent the median and interquartile range (IQR), error bars represent the range, and dots denote outliers.

**Fig. 3:**
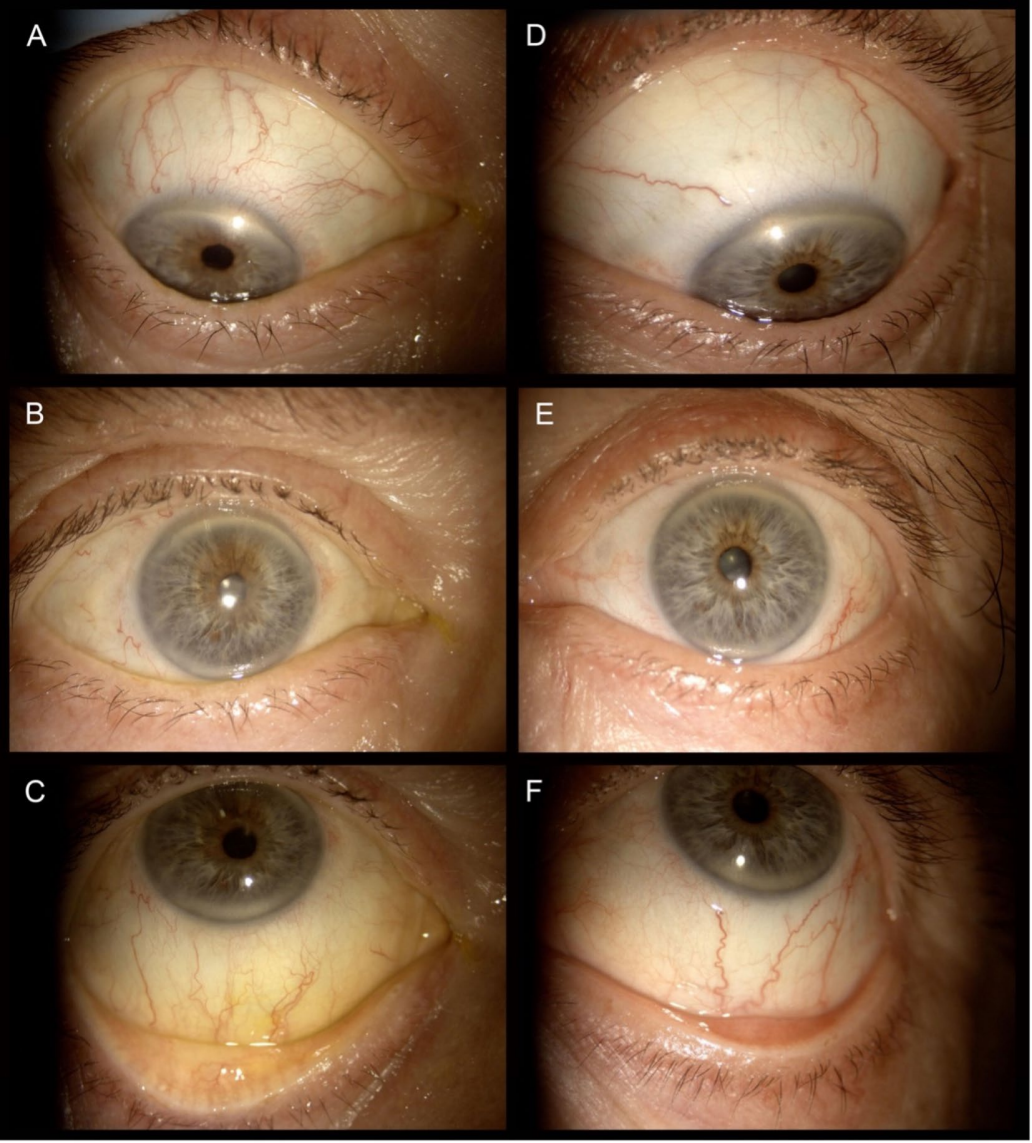
Slit lamp pictures of the ocular surface in a representative glaucomatous patient with GT-OSD, which show a higher hyperemia in the untreated fellow eye (right eye, A-C) compared to the treated eye (left eye, D-F).

Ocular surface parameters in TE and FE are reported in Table 2. Overall, the median BRS was significantly lower in TE concerning FE (*p* = 0.011), with conjunctival hyperemia approximatively 14% lower in eyes receiving aflibercept (Fig. 2, A). When analyzing median fluorescein TBUT, values were significantly higher in TE compared to FE (*p* = 0.037), which means approximately a 10% greater stability of the tear film (Fig. 2, B). Conversely, we did not find any significant differences for NIBUT, ST, CFS, and lower eyelid MG dropout between TE and FE. However, despite CFS and NIBUT did not significantly differ between eyes, their median values showed a marginally tendency for better results in TE compared to FE. Inter-eye TMH difference was very close to reach the statistical significance. Figure 3 shows slit lamp pictures of the ocular surface in patients treated with aflibercept, in which TE presents a more contained hyperemia compared to the untreated eye.

**Table 2:** Ocular surface parameters in TE and FE.

Ocular surface parameter	TE	FE	Wilcoxon U test *p*-value
BR SCORE *(EFRON scale 0–4)*	2 *(1–2)*	2 *(1–3)*	***0.011***
CFS *(OXFORD scale 0–5)*	1 *(0–1.75)*	1 *(0–2)*	*0.412*
NIBUT *(sec)*	6.7 *(4.7–10.6)*	6.6 *(4.0–9.4)*	*0.102*
Fluorescein TBUT *(sec)*	6.0 *(3.0–9.0)*	4.5 *(3.1–8.7)*	***0.037***
TMH *(mm)*	0.4 *(0.3–0.4)*	0.4 *(0.2–0.4)*	***0.050***
LOWER EYELID MG LOSS *(%)*	21 *(15–29)*	23 *(10–33)*	*0.990*
ST I *(mm)*	10.0 *(5.0–12.0)*	7.5 *(5.0–10.0)*	*0.155*

TE: treated eye; FE: fellow eye; BR: Bulbar Redness; NIBUT: Noninvasive Break-Up-Time; TBUT: tear film break- up time; TMH: Tear meniscus height; MG: Meibomian glands. CFS: corneal fluoresceine staining; STI: Schirmer test I. Data are expressed as median and interquartile range (IQR) reported as first and third quartile.

When analyzing correlations between the number of IVIs and all the ocular surface parameters, no significant associations were found in the exploratory analysis. Significant correlations were only found between the number of IOP-lowering eyedrops and BR (rho = 0.45 (*p* = 0.022)) and CFS (rho = 0.46 (*p* = 0.019)), and between the number of IVIs and age, (rho = 0.41 (*p* = 0.019)).

## Discussion

Given its high prevalence, GT-OSD can be considered an impactful concomitant disease of glaucoma, further decreasing patients QoL and forcing clinicians to adopt parallel management in most cases^7^. Unfortunately at least 15% of patients with glaucoma may also develop vision-threatening retinal comorbidities such as DME, RVO, and n-AMD, which currently represent the main indications to IVIs of anti-VEGFs^12,13^. Thus, glaucomatous patients could eventually receive different concomitant treatments potentially resulting in unexpected effects between them.

In the present study we found that patients with a moderate to severe form of GT-OSD and retinal comorbidities, presented better ocular surface conditions in the eye that received serial IVIs of aflibercept, compared with the fellow untreated eye. This was supported by a reduced conjunctival redness and an improved TF stability (higher TBUT).

These results are in line, though not entirely, with a recent similar study which analyzed the ocular surface changes in patients with dry eye undergoing IVIs of anti-VEGF for n-AMD^14^. In fact, while the bulbar redness reduced in both studies, the MG dropout improved only in dry eye, whereas TBUT improved only in glaucoma. Although without statistical significance, NIBUT showed a tendency toward healthier values in TE compared to FE in both studies. TMH, a robust biomarker of dry eye, improved clearly in the study of Malmin et al., but remained at limit of the statistical significance in the present one (Table 2).

Some reasons could underlie the partial differences between studies. The first one is probably linked to the number of IVIs that received the two samples, since in the study of Malmin et al. dry eye patients underwent a significantly higher median number of IVIs (19.5) compared to our study^14^. This could have limited the effects of anti-VEGFs in patients with glaucoma, allowing improvements only on the conjunctival vasculature (BR) and the mucosal components of the tear film (TBUT), but not on MGs. Probably a higher number of IVIs of aflibercept, because of a presumed cumulative effect, would have induced favorable changes also to MGs in patients with GT-OSD. However, since we did not analyze the upper eyelid, we cannot completely exclude the presence of some positive modifications of MGs also in our study. Second, GT-OSD is an iatrogenic/toxic form of dry eye, characterized by a prevalently obstructive form of MGs dysfunction^3^. Thus, different anatomo-pathological conditions of MGs between dry eye and glaucoma, could underlie a different sensibility of MGs to the anti-VEGF injection procedures. Third, patients with glaucoma continued to instill IOP lowering eyedrops after each IVIs, without any interruption, and received topical unpreserved antibiotics as post-procedure therapy for few days. Fourth, the favorable anti-microbial effects of anti-septic eyedrops (PVP-I) on the composition of eyelid commensals, which has been proposed as one the possible reasons for the MGs dropout reduction in dry eye, could be different in patients with glaucoma. As known, the ocular surface microbiota is profoundly modified in these patients, due to the effects of IOP lowering medications or the presence of systemic comorbidity^18^. Given that, we may hypothesize that the antiseptic-induced modifications on eyelid commensals in glaucoma, probably because of an unfavorable imbalance among micro-organisms, could not be useful to improve at best the ocular surface. Further studies dedicated to specifically evaluating the role of the anti-septic eyedrops in optimizing the ocular surface in glaucoma are requested to confirm our speculations. Finally, one cannot rule out that, since the two studies utilized slightly different methodologies to evaluate the ocular surface (Sirius corneal topographer/tomographer vs. Keratograph 5 M), results could not completely match. Thus, because of all these potential confounding factors, some of the beneficial effects of the IVI procedure could be antagonized or masked.

These hypotheses could be also considered when questioning the absence of differences between TE and FE concerning the other parameters. TMH, whose inter-eye differences was very close to the statistical significance in our study, has been proposed to increase in dry eye after repeated IVIs as a response to the epitheliopathy induced by PVP-I (which probably stimulate the main lacrimal gland secretion)^8,19,20^. Though we observed worse CFS score compared to Malmin’s et al. results, there could have been other unidentified factors, such as differences in the efficiency of the lacrimal drainage system between the two samples that may explain the limited improvement of TMH in our study^14^.

The improvement of conjunctival hyperemia represents an important feature since, as BR score is a marker of inflammation, it suggests that IVIs have anti-inflammatory properties on the ocular surface in both dry eye and GT-OSD^21–23^. The effects on tear film stability and, thus, on TBUT are not completely clear. In fact, though on one side our results differed from those of Malmin et al., on the other side agreed with a study reporting increased TBUT values after direct anti-VEGF injections inside MGs^14^. One may hypothesize that differences may depend, at least in part, on the conditions of the main lacrimal gland and the mucin producing components of the ocular surface, the number of IVIs, and on the site of administration of the anti-VEGF. Conversely, we cannot state whether different anti-VEGFs exert different effects.

Concerning mechanisms inducing such modifications, mixed effects of PVP-I and aflibercept can be claimed. As stated above PVP-I, because of its antiseptic properties, could remodulate the microbial flora disfavoring some micro-organisms, such as the Demodex Folliculorum, which are involved in the MG dysfunction^24^.

On the other hand, anti-VEGFs may reduce the higher levels of VEGFs contained within tears of patients with glaucoma^25^. Since VEGFs are well established inflammatory mediators, the reduction of their concentrations may lead to several anti-inflammatory effects, such as the reduction of the bulbar hyperemia and the mitigation of dry eye.

Some limitations must be pointed out. First of all, the present study had a limited sample size and patients received a low number of injections: these aspects could have masked some effects of the procedure on GT-OSD and doesn’t allow to give our results a clear clinical relevance. Second, we enrolled only patients that received aflibercept: thus, we cannot state whether results obtained are generalizable to other anti-VEGFs or not. Further studies comparing the effects of all currently utilized anti-VEGFs are required. Third, more performing diagnostic technologies, such as confocal microscopy or immune-cytology, would have probably permitted to unravel in a more sensitive way some of the IVIs-induced changes or to reveal additional findings. Finally, the present study is retrospective and observational and, thus, does not enable us to establish causality between IVIs and GT-OSD changes. Further prospective, long-term studies considering patients who underwent a higher number of IVIs, who used all the commercially available anti-VEGFs, and with a longer follow-up, are required to confirm these preliminary data and to evaluate whether modifications of the ocular surface induced by the anti-VEGF administration improve also the QoL.

In closing, our preliminary results, are in line with previously reported positive effects of anti-VEGFs or topical antiseptics on the ocular surface in patients with OSD. Whether confirmed in larger prospective studies, these findings could be of particular interest since may open to unexplored strategies to manage the GT-OSD.

## Data Availability

The datasets generated during and/or analyzed during the current study are available from the corresponding author on reasonable request.
